# Wheeze in Preschool Age Is Associated with Pulmonary Bacterial Infection and Resolves after Antibiotic Therapy

**DOI:** 10.1371/journal.pone.0027913

**Published:** 2011-11-29

**Authors:** Nicolaus Schwerk, Folke Brinkmann, Bisharah Soudah, Michael Kabesch, Gesine Hansen

**Affiliations:** 1 Department of Pneumology, Allergy and Neonatology, University Children's Hospital, Hanover Medical School, Hanover, Germany; 2 Institute of Pathology, Hanover Medical School, Hanover, Germany; University of Tübingen, Germany

## Abstract

**Background:**

Neonates with airways colonized by *Haemophilus influenzae*, *Streptococcus pneumoniae* or *Moraxella catarrhalis* are at increased risk for recurrent wheeze which may resemble asthma early in life. It is not clear whether chronic colonization by these pathogens is causative for severe persistent wheeze in some preschool children and whether these children might benefit from antibiotic treatment. We assessed the relevance of bacterial colonization and chronic airway infection in preschool children with severe persistent wheezing and evaluated the outcome of long-time antibiotic treatment on the clinical course in such children.

**Methodology/Principal Findings:**

Preschool children (n = 42) with severe persistent wheeze but no symptoms of acute pulmonary infection were investigated by bronchoscopy and bronchoalveolar lavage (BAL). Differential cell counts and microbiological and virological analyses were performed on BAL samples. Patients diagnosed with bacterial infection were treated with antibiotics for 2–16 weeks (n = 29). A modified ISAAC questionnaire was used for follow-up assessment of children at least 6 months after bronchoscopy. Of the 42 children with severe wheezing, 34 (81%) showed a neutrophilic inflammation and 20 (59%) of this subgroup had elevated bacterial counts (≥10^4^ colony forming units per milliliter) suggesting infection. *Haemophilus influenzae*, *Streptococcus pneumoniae* and *Moraxella catarrhalis* were the most frequently isolated species. After treatment with appropriate antibiotics 92% of patients showed a marked improvement of symptoms upon follow-up examination.

**Conclusions/Significance:**

Chronic bacterial infections are relevant in a subgroup of preschool children with persistent wheezing and such children benefit significantly from antibiotic therapy.

## Introduction

Persistent wheezing in early childhood is a common but insufficiently characterized condition. One in three children has been reported to experience at least one episode of wheezing during the first 3 years of life [1]–[3]. Preschool children with persistent wheezing frequently respond poorly to conventional asthma therapy and management of persistent symptoms is difficult and costly.

So far, mainly viral infections have been considered trigger factors for early childhood wheezing and suggested to even be causally involved in the development of recurrent wheezing and asthma. Only recently a correlation has been observed between colonisation of the upper airways with *Haemophilus influenzae*, *Streptococcus pneumoniae* or *Moraxella catarrhalis* during the first months of life and the subsequent development of asthma [4]. Interestingly, a similar pattern of microbial colonisation was found in the lower airways of children with asthma, but not in healthy individuals in a recent study using advanced technology of bacterial 16S rRNA sequencing [5].

The clinical significance and impact of these observations remains unclear. Are the identified bacterial species found in the airways an epiphenomenon or the cause of a subform of preschool wheezing that has not yet been discriminated sufficiently from asthma? In the latter case sufficient antibiotic therapy would be the treatment of choice in children with persistent wheezing and significant isolates of *H. influenzae*, *S. pneumoniae or M. catarrhalis* from the lungs. Thus, we explored the presence of bacteria and viruses in the airways of preschool children with recurrent, severe persistent wheezing who had been diagnosed with asthma according to current clinical standards such as the PRACTALL guidelines [6]. We investigated the effect of adequate antibiotic treatment on the persistence of wheezing and other pulmonary symptoms in children from whom relevant bacterial counts and neutrophilic inflammation were detected in the BAL fluid.

## Materials and Methods

### Objectives

We assessed the relevance of bacterial colonization and chronic airway infection in preschool children with severe persistent wheezing and evaluated the outcome of long-time antibiotic treatment on the clinical course in such children.

### Patients

In this retrospective study a total of 98 children under the age of six years who underwent flexible bronchoscopy for severe recurrent or persistent wheeze were recruited between 2003 and 2010 at our institution. Only children with more than two episodes of wheeze in the 6 months prior to testing and wheezing in the absence of any signs of infections were included. Prematurely born infants, those with other pulmonary pathologies and children with isolated wet cough were excluded from the analysis. No child included was on antibiotic therapy or displayed any signs of acute infection at the time of bronchoscopy. Further patients' characteristics are listed in Table 1.

**Table 1:** Patients' characteristics.

	Patients (n = 42)
Females	21 (50%)
Median age in months (range)	30,5 (5–67)
Number of hospital admission (range)	1,4 (0–10)
Doctor's diagnosis of asthma	31 (74%)
Diagnosis of asthma*	42 (100%)
Family history of asthma/atopy**	19 (48%)
Previous hospital admission because of wheeze	26 (63%)
**Treatment:**	
Inhaled steroids ever	37 (88%)
Inhaled steroids at time of investigation	29 (69%)
Inhaled beta agonists ever	42 (100%)
Leucotriene antagonists ever	20 (47%)

*According to PRACTALL criteria.

**Data on family history was available for 39 patients only.

### Reference group for BAL findings (Control group)

14 children without wheeze who underwent bronchoscopy for the investigation of various pulmonary diseases (Table 2) served as a reference group for bronchoalveolar lavage findings. They were not included in the follow-up.

**Table 2:** Controls' characteristics.

	Controls (n = 14)
Females	4 (29%)
Median age in months (range)	12 (1–64)
Exclusion tracheal/bronchial malformation	7 (50%)
Foreign body aspiration/ Aspiration	5 (36%)
Atelectasis	2 (14%)

### Laboratory investigations

Quantitative pilocarpine iontopheresis, assessment of immunoglobulines including total IgE levels, and measurement of specific IgE to inhalant and/or nutritional allergens were conducted in all patients.

### Radiology

Chest X-ray was performed on all patients but not routinely on controls.

### Bronchoscopy and bronchoalveolar lavage

Flexible bronchoscopy was performed using a 2,8 mm or 3,8 mm diameter bronchoscope (BF-XP160F, BF-3C160, Olympus, Key-Med, Southend-on-Sea, UK) depending on the child's size. After inspection of the upper and lower airways a BAL using sterile 0.9% saline solution (3×1 ml/kg) was performed at the side on which inflammation had been visually identified, or on the middle lobe.

### Microbiological and viral analysis

The first aliquot of BAL fluid was used for microbiological analysis. Quantitative and qualitative bacterial cultures were conducted using standard methods. A positive bacterial culture was defined as growth of a single bacteria species of ≥10^4^ cfu/ml BAL [7].

The second BAL aliquot was employed for viral analysis using an immunofluorescence testing including influenza A and B, parainfluenza types 1–3, herpes simplex, rhinovirus, metapneumovirus, respiratory syncytial virus and adenovirus.

### Cytology

The third BAL aliquot was used for cytological analysis. Samples were diluted in sodium sulfate (1∶1) and stained with Pappenheim, Papanicolaou, Sudan, Berlin Blau and periodic acid- Schiff reagents. Neutrophilia was defined as the presence of >2 neutrophils, eosinophila as >1 eosinophils and lymphocytosis as >10 lymphocytes per 100 BAL cells. Fat-laden macrophages were stained with Sudan reagent and samples with >5% of such cells were considered to be pathologic [8].

Colonization was determined as the presence of bacteria without clinical or cytological signs of infection (no elevated neutrophil count in the BAL) while inflammation relates to the presence of elevated neutrophil/eosinophil or lymphocyte count in the BAL. Infection was defined as the presence of bacterial count ≥10^4^ cfu/ml of at least one single species and inflammation in the BAL and clinical signs of chronic infection (like persistent wheeze). The cut- off for bacteria we considered to be relevant is ≥10^4^ cfu/ml according to the literature [7].

### Follow- up

Standardized telephone questionnaires' were applied to record the follow up. Questionnaires regarding the use of prior antiasthmatic and antibiotic therapy, hospital admissions and conduct of follow-up at least 6 months after bronchoscopy were used for the assess of all patients. Asthma symptoms were based on ISAAC core questions [9].

### Ethics

The study protocol of this retrospective analysis was approved by the Ethical Committee of Hannover Medical School. Verbal consent was obtained from the parents of all children who participated in the follow- up questionnaire and stated on the questionnaire. No further consent was requested by the local Ethical Committee.

### Statistical Analysis

Data were analysed using SPSS 17.0 statistics software. Comparisons between patient and control group in regard to inflammation and bacterial count were made using non parametric tests (Mann-Whitney U Test). For association between categorical variables in the follow-up Chi-square test or Fisher's exact test was performed. A p -value less than 0,05 was considered to be statistically significant.

## Results

56 out of 98 patients had to be excluded due to airway malformation, prematurity or acute signs of infection (fever, elevated inflammatory markers) at the date of bronchoscopy. A total of 42 patients with severe recurrent or persistent wheezing without any signs of acute infections were included in the study. All fulfilled the criteria for asthma according to the PRACTALL guidelines [6]. Most patients (88%) had been treated with inhaled corticosteroids (200–400 µg budesonide equivalent) for an average of 20,2 weeks before bronchoscopy; mostly without clinical improvement (86%). There was no difference neither in ethnical background or living environment between patients and controls. Both populations were predominantly of Caucasian origin, about 25% of the study population and 15% of the control group of Mediterranean decent (Turkish, Italian). The majority (>85%) of patients and controls lived in an urban environment. Further patients' characteristics are listed in Table 1, the results of laboratory examinations in Table 3.

**Table 3:** Results of clinical investigations (patients only).

Variables	Laboratory investigations performed (n = 42)	Patients with abnormalities*
Sweat test	42	0
Specific IgE	42	10 (24%)
Total IgE	42	12 (29%)
IgG, IgA, IgM	34	0 (0%)

*Values are given as No. (%).

All 42 patients and 14 controls underwent flexible bronchoscopy and BAL. The results of the investigations are shown in Table 4.

**Table 4:** Microbiological differentiation of bronchoalveolar lavage.

	No. patients(n = 42)	No. controls(n = 14)
bacterial count ≥10^4^ cfu/mlwith neutrophilia	22 (52%)20/22 (91%)	3 (21%)0/3 (0)
H. influenzae	13 (31%)	0
Streptococcus pneumoniae	8 (19%)	0
Moraxella catarrhalis	4 (10%)	0
Staphylococcus aureus	3 (7%)	0
Other bacteria	2 (5%)	3 (21%)
Viral infection	2 (5%)	0

**Figure 1:**
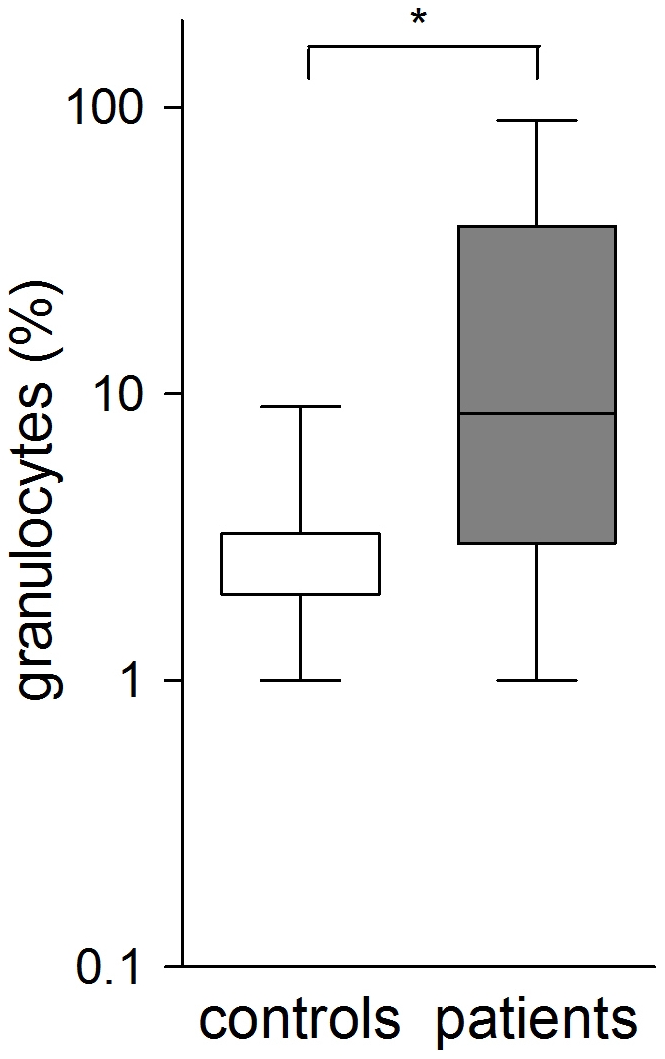
Comparison of granulocyte and bacterial count in the bronchoalveolar lavage fluid of patients, number of patients: n = 42, significance levels were calculated using Mann- Whitney- U- Test (p<0,005).

**Figure 2:**
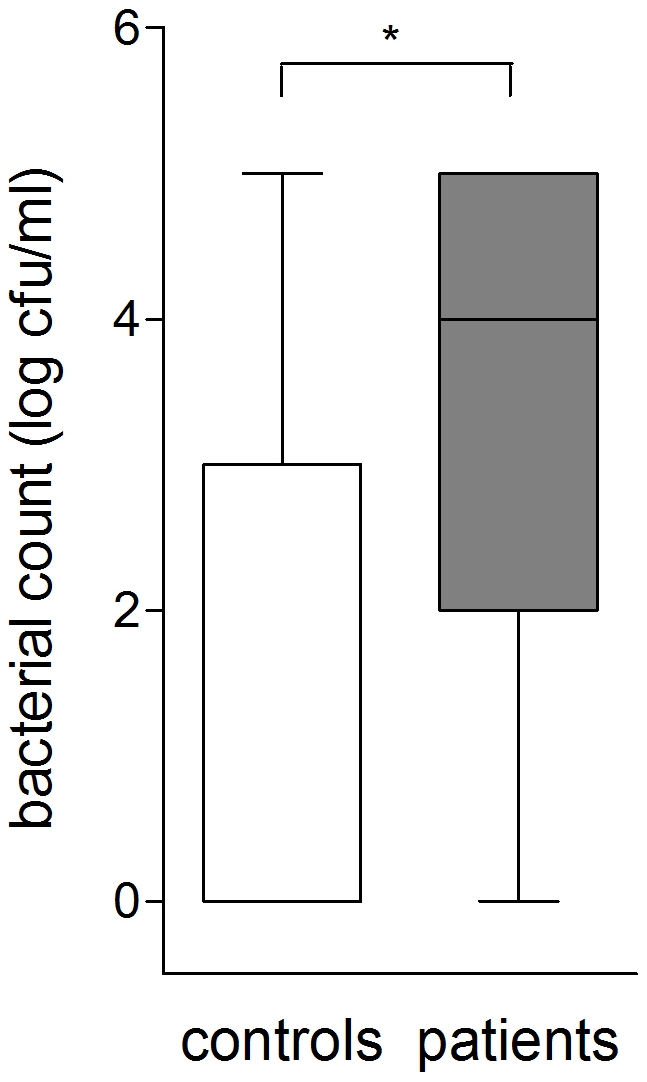
Comparison of granulocyte and bacterial count in the bronchoalveolar lavage fluid of controls, number of controls: n = 14, significance levels were calculated using Mann- Whitney- U- Test (p<0,005).

**Figure 3:**
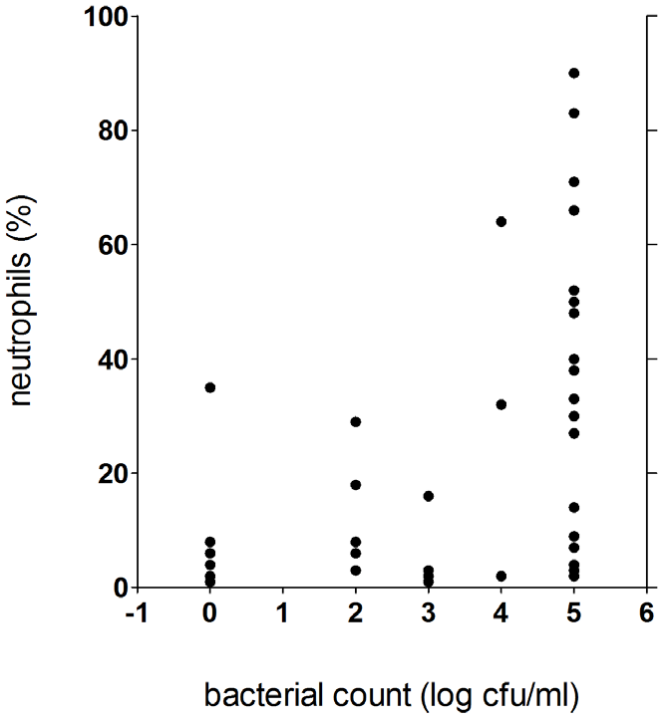
Correlation between bacterial and neutrophilic count in the bronchoalveolar lavage fluid of patients, number of patients: n = 42, each dot represents a patient, 5 dots overlap, significance levels were calculated using Mann- Whitney- U- Test (p<0,005).

**Figure 4:**
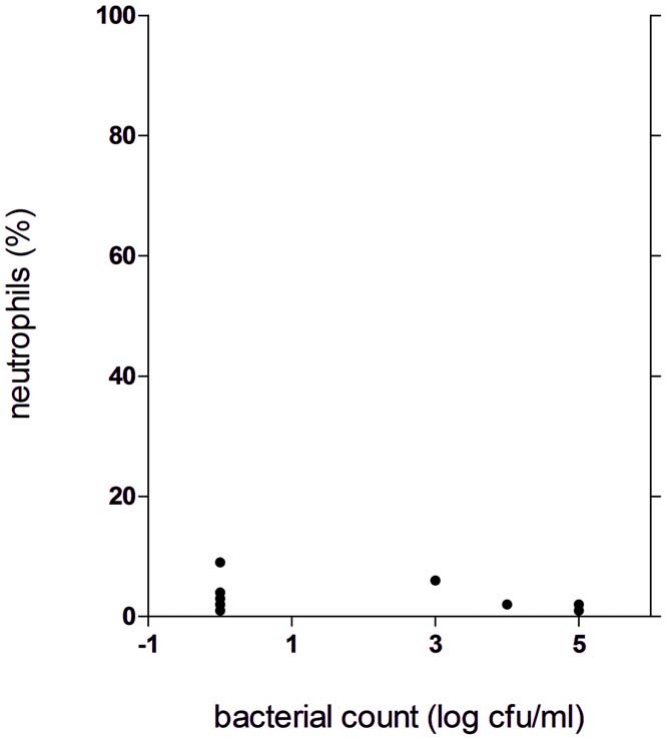
Correlation between bacterial and neutrophilic count in the bronchoalveolar lavage fluid of controls, number of controls: n = 14, each dot represents a control, significance levels were calculated using Mann- Whitney- U- Test (p>0,05).

**Figure 5:**
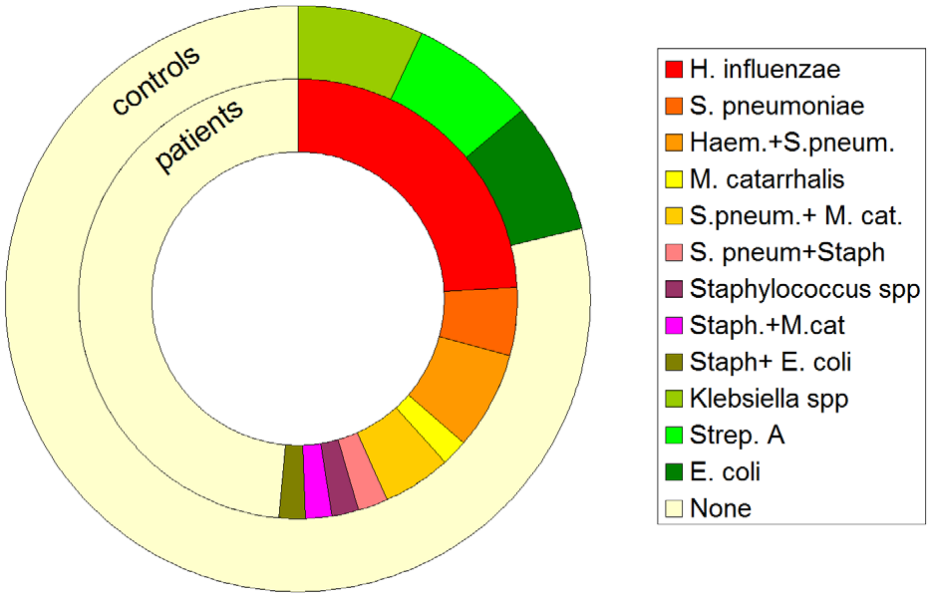
Percentage of patients and controls with bacterial count ≥10^4^ cfu/ml per different bacterial species. Inner Ring: patients (n = 42), outer ring: controls (n = 14).

Significant increase in both neutrophilia and bacterial count were observed in patients compared to the control group (p<0.005) using nonparametric tests (Fig. 1, 2). An increase of bacterial cell count above 10^4^ cfu/ml and BAL-neutrophilia was considered as relevant bacterial infection. The levels of neutrophils in the BAL fluid were significantly higher in the subgroup of patients with bacterial cell count ≥10 ^4^ cfu/ml than in those with lower cell counts (Fig. 3) using nonparametric tests. Seven controls displayed either significant bacterial counts or neutrophilia but not both (Fig. 4). Bacteria species cultivated from the patients consisted mostly of *H. influenzae, S. pneumoniae and M.catarrhalis*, whereas none of the controls were colonized with these bacteria. Instead *Klebsiella sp.* and *Enterobacter spp.* were isolated from controls (Fig. 5 and Table 4). Viral infection was detected in two patients (RS Virus and parainfluenza Type II), one of them also showing growth of *H. influenzae* and neutrophilic inflammation. Eosinophilia was detected in five patients (12%) and elevated numbers of fat-laden macrophages were found in four patients (10%) but in none of the controls (see supplementary table S1).

At the time of bronchoscopy 37 out of the 42 patients received a chest x-ray. 31 did not show any pathological findings except hyperinflation. Three patients showed atelectatic changes. Infiltrates were seen in another three patients in absence of any signs of clinical infection. These patients had neutrophilic inflammation in the BAL, in two cases with relevant bacterial counts. In five patients chest x-ray was not done at the time of bronchoscopy but during exacerbations more than four weeks ago and showed infiltrates at that point in time. Three of those patients had neutrophilic inflammation and elevated bacterial counts while two displayed no sign of inflammation in the BAL.

All patients with both neutrophilia and an elevated bacterial count (≥10^4^ cfu/ml) were considered to have a relevant lower respiratory tract infection (n = 20) and received antibiotic treatment. Five children with and four children without neutrophilia who had lower bacterial counts (<10^4^ cfu/ml) also received antibiotic treatment because of bronchoscopic signs of a relevant chronic inflammation. Thus, a total of 29 children received antibiotic therapy with amoxicillin (n = 17), amoxicillin and clavulanic acid (n = 4), cefuroxim (n = 4) or trimethoprim and sulfamethoxazole (cotrimoxazole) (n = 4) for a median of six weeks (range 2–16 weeks).

**Figure 6:**
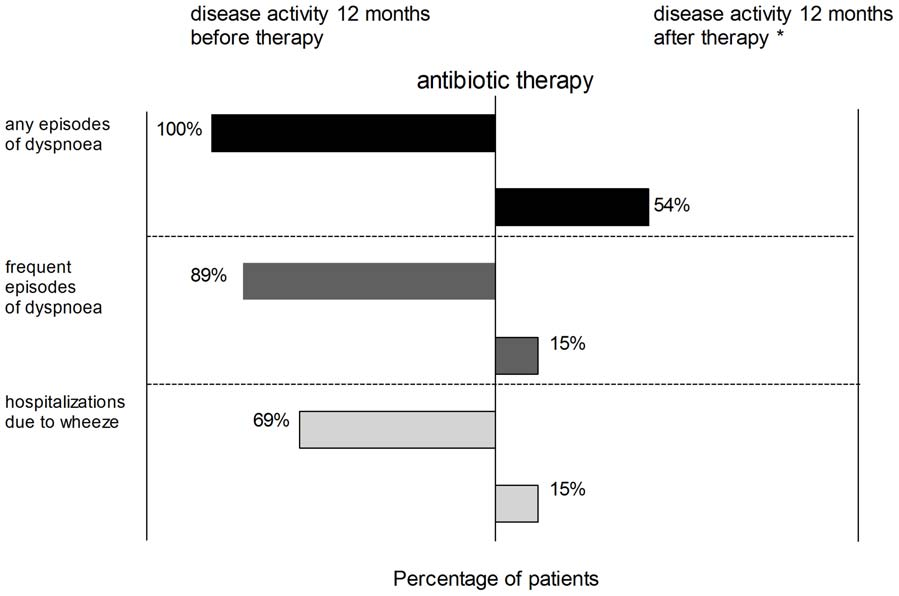
Disease activity of patients before and after antibiotic therapy. Number of patients: n = 24, significance levels were calculated chi square/ Fisher's exact test (p<0,005 for all parameters) * three patients could only be followed up for 6, 8, 10 months.

Of the 42 patients 35 were followed up for at least six months (median 32 months, range 6–67 months), seven were not available for follow up. Out of the 35 patients in follow-up 26 received antibiotic therapy; the remaining nine continued on anti-asthmatic medication only. These two groups did not differ in age (mean 31 vs 33 months), prevalence of sensitization (33 vs. 31%) or follow-up period (mean 31,6 vs 34,4 months). In the group of patients treated with antibiotics, 24 out of 26 (92%) improved after therapy (showing fewer symptoms and exacerbations according to the ISAAC questionnaire). In 81% the improvement persisted for more than six 6 months and 65% either reduced or discontinued asthma medication. Only 14 out of 26 (54%) children reported any episodes of dyspnoea caused by wheeze in the follow up after antibiotic treatment (p<0,005, chi-square test). The rate of children requiring hospitalization because of severe wheeze dropped from 69% in the year preceding therapy to only 15% in the follow-up period (18 vs 4 patients, p<0,005, chi square test) as shown in Figure 6. In the group that did not receive antibiotic therapy only one out of nine children could reduce asthma medication. In two patients episodes of dyspnoea became less frequent and hospitalization rate decreased from 89% to 44% after bronchoscopy.

## Discussion

The present study showed that neutrophilia and significant bacterial counts can be detected by bronchoalveolar lavage of the lower respiratory tract in a relevant number of preschool children diagnosed with childhood asthma. The most frequently isolated species include *Haemophilus influenzae*, *Streptococcus pneumoniae* and *Moraxella catarrhalis*. Further, we showed that most patients improved significantly after appropriate antibiotic therapy and that the improvement persisted in terms of both symptom reduction and less exacerbations.

Our study population did not differ in either demographic characteristics or the frequency of atopy from other asthma populations in the same age range [10]. All patients met the criteria for preschool asthma according to the PRACTALL 2008 guidelines [6], and had multi-trigger wheezing, as defined in the ERS guidelines [11]. Both guidelines recommend that inhaled steroids and/or oral leucotriene antagonists be given to children with persistent symptoms. Consequently, almost all patients in our study had received asthma medication for a considerable time before admission. However, most patients responded poorly to such medication and were therefore admitted for further investigation.

Changes in the chest x-ray like atelectasis or infiltrates have been described previously in systematic studies of adults and children with severe asthma [12]–[14]. In our patients chest X-ray findings did not correlate well with clinical symptoms and only some of these children showed neutrophilic inflammation and elevated bacterial counts in the BAL, suggesting that chest x-ray is not sufficiently sensitive to identify chronic bacterial infection in this population.

It might be argued that the children in our study did not have asthma but rather persistent bacterial bronchitis which can mimic a form of asthma that is difficult to treat. In a study of 81 children with persistent bacterial bronchitis, 58% had received ineffective asthma treatment previous to diagnosis. Almost 50% were reported to wheeze although this symptom could not be verified in most of the patients under study conditions [15]. A chronic wet cough is pathognomonic for patients with persistent bacterial bronchitis. As children with chronic wet cough had specifically been excluded from our study, any misclassification of our patients with persistent bacterial bronchitis is unlikely and cannot easily explain our findings.

In most of our patients we observed elevated numbers of neutrophils in the BAL fluid as a possible sign of inflammation. This finding alone does not necessarily indicate the presence of a clinically relevant bacterial infection as high neutrophil counts have also been observed in healthy preschool children [16]. However, neutrophilic inflammation seems to be related to wheezing in young children because even in wheezy children that did not yield positive bacterial cultures, the neutrophil count was still higher than in the control group [17].

The bacteria most frequently cultivated by standard techniques in our study were *H. influenzae*, *S. pneumoniae* and *M. catarrhalis*. In contrast, we did not isolate any of these species from lavage fluid of the control group, indicating that children without wheeze do not harbour a relevant number of such bacteria in the lower airways. It might be argued that the bacteria that were successfully cultivated may not be those responsible for the chronic inflammation of the lower airways.

We did not specifically test for atypical bacteria such as *Mycoplasma* or *Chlamydia pneumoniae* in our patients although these had been suggested previously to contribute to asthma or asthma exacerbations [18], [19]. In a retrospective study on 39 children who underwent extensive examinations because of recurrent wheeze no case of acute *Mycoplasma* or *Chlamydia* infection was identified [20] and it may be assumed that infections with atypical bacteria are not relevant in the age group we studied. Also, we did not employ a microbiome analysis by 16S rRNA sequencing to explore the possible presence of bacteria not easily detected by standard culturing methods. Interestingly, a recent study in asthmatic adults and children using 16S rRNA sequencing techniques also identified *H. influenzae* as the most frequent isolate in patients: this strain was rarely found in healthy controls [5].

The combination of clinical symptoms, neutrophilic inflammation and positive bacterial culture in our patients suggests relevant infection of the lower respiratory tract [21] with the isolated bacteria and justified the use of antibiotic therapy. The fact that the children responded so well to antibiotic therapy aimed at the isolated bacteria narrows down the range of bacterial species that may have been responsible for the infection and the wheezing.

Most of our patients that showed neutrophilic inflammation but who had bacterial counts below 10 ^4^ cfu/ml were not treated with antibiotics. Whether that cut off for a bacterial count from the airways is clinically relevant needs to be discussed as no clear data on physiological bacterial flora in the airways of children exists. Thus, it may be speculated that bacterial counts below 10^4^ cfu/ml could also cause significant endobronchial inflammation. Consequently, preschool children with severe asthma like symptoms and neutrophilic inflammation in lavage fluid may also benefit from antibiotic therapy even if the bacterial cell counts are below 10^4^ cfu/ml. Further studies to determine an appropriate threshold for treatment are needed.

In the present work we treated patients with antibiotics for a median of six weeks. In the absence of guidelines for the duration of antibiotic treatment of chronic lung infections in children we prescribed long-term antibiotic therapy similar to that suggested by several authors for treatment of chronic bacterial lower respiratory tract infections in children [22], [23]. This approach was successful. However, it is not clear if a treatment for only seven days as suggested in a recent Cochrane review for moist cough [23] would not be equally effective.

At this point, it is unknown whether bacterial colonization with *H. influenzae* or any of the other index bacteria reoccurs after cessation of therapy. While a second bronchoscopy and BAL is difficult to justify in a child free of symptoms, assessment of bacterial colonization of the airways by induced sputum might be an option in children older than those included in our study.

Finally, the question remains whether colonization of the airways with *H. influenzae* or other pathogens is caused by mucosal immunodeficiency or whether this is in fact the primary cause of chronic inflammation in a subgroup of children with symptoms of persistent wheezing. It may be argued that local immunodeficiency could be caused by inhaled steroids which most of the children received at the time of investigation. However, two recent reports describe the early colonization of the airways with *H. influenzae*, *S. pneumoniae* and *M. catarrhalis* preceding any development and treatment of asthma. These very same species were found upon later asthma exacerbations in the same population [4], [24]. Thus, colonization by such bacteria seems to occur very early in life, before any medications has been started and may reflect the existence of host specific susceptibility factors for airway colonization by specific microbes.

This retrospective study included only a small number of patients and controls. Although the findings strongly support the relevance of lower airway inflammation and infection in these children, the heterogeneity of the patient and control population in terms of age, gender and follow-up limits the predictive power of these observations.

Furthermore, in this study the assessment of bacteria is based just on standard techniques. The observation that the children responded well to a targeted but simple antibiotic therapy is nevertheless intriguing and warrants further investigation using prospective well designed cohort studies. We conclude that there is a great need for more precise phenotyping of the asthma syndrome seen in early childhood. While all children in our study fulfilled the criteria for the diagnosis of asthma as defined in the PRACTALL guidelines [6], these children did not respond well to asthma therapy. Instead they were found to suffer from clinically relevant bacterial infection. Thus, we suggest that antibiotic therapy might be a valuable treatment option in a subgroup of children so far classified as asthmatics. The effectiveness and the necessary duration of such treatment need to be further assessed.

## Supporting Information

Table S1 **Cellular components of bronchoalveolar lavage.**
(DOC)
