# Supplementary material for: Wheeze in Preschool Age Is Associated with Pulmonary Bacterial Infection and Resolves after Antibiotic Therapy
Source: PLoS One. 2011 Nov 29;6(11):e27913. doi: 10.1371/journal.pone.0027913 (PMC3226624; doi:10.1371/journal.pone.0027913)
Supplement: Table S1 — Cellular components of bronchoalveolar lavage. (DOC) [file pone.0027913.s001.doc]

**Table S1**

**Cellular components of bronchoalveolar lavage**

| **Differential Cellular Count** | **Reference Range^*^** | **No. patients**  **(% )** | **Patients Mean, (range)** | **No. controls (%)** | **Controls mean, (range)** | ***p-value*** |
| --- | --- | --- | --- | --- | --- | --- |
| Neutrophilia  With bacterial count >=10^4^ | 0-2% | 34 (81)  20/34 (59) | 23,9  (1-90) | 4 (20)  0/4 (0) | 2,9  (0-9) | 0,004 |
| Lymphocytosis  With bacterial count >=10^4^ | 4-10% | 8 (19) | 7,9  (1-22) | 1 (7) | 6,1  (4-12) | NS |
| Eosinophilia | 0% | 5 (12) | 1,4 (0-31) | 1 (7) | 0,07 (0-1) | Ns |
| Multiple cell lines increased |  | 11 (26) |  | 1 (7) |  | <0,001 |

^*^Reference range is the percentage of all cells in BAL fluid
